# Activation of Meningeal Afferents Relevant to Trigeminal Headache Pain after Photothrombotic Stroke Lesion: A Pilot Study in Mice

**DOI:** 10.3390/ijms232012590

**Published:** 2022-10-20

**Authors:** Georgii Krivoshein, Abdulhameed Bakreen, Arn M. J. M. van den Maagdenberg, Tarja Malm, Rashid Giniatullin, Jukka Jolkkonen

**Affiliations:** 1 A.I. Virtanen Institute for Molecular Sciences, University of Eastern Finland, 70211 Kuopio, Finland; 2 Department of Human Genetics, Leiden University Medical Center, 2300 RC Leiden, The Netherlands; 3 Department of Neurology, Leiden University Medical Center, 2300 RC Leiden, The Netherlands

**Keywords:** headache, meninges, mice, nociception, pain, photothrombosis, Piezo1, stroke

## Abstract

Stroke can be followed by immediate severe headaches. As headaches are initiated by the activation of trigeminal meningeal afferents, we assessed changes in the activity of meningeal afferents in mice subjected to cortical photothrombosis. Cortical photothrombosis induced ipsilateral lesions of variable sizes that were associated with contralateral sensorimotor impairment. Nociceptive firing of mechanosensitive Piezo1 channels, activated by the agonist Yoda1, was increased in meningeal afferents in the ischemic hemispheres. These meningeal afferents also had a higher maximal spike frequency at baseline and during activation of the mechanosensitive Piezo1 channel by Yoda1. Moreover, in these meningeal afferents, nociceptive firing was active during the entire induction of transient receptor potential vanilloid 1 (TRPV1) channels by capsaicin. No such activation was observed on the contralateral hemi-skulls of the same group of mice or in control mice. Our data suggest the involvement of mechanosensitive Piezo1 channels capable of maintaining high-frequency spiking activity and of nociceptive TRPV1 channels in trigeminal headache pain responses after experimental ischemic stroke in mice.

## 1. Introduction

Stroke is the second most common cause of death and the leading cause of disability in adults in the European Union [1]. Acute neurological complications of a stroke, such as headaches, likely contribute to whether patients fully recover or suffer from persistent functional disability [2], but the underlying mechanisms are unclear.

Diagnostic criteria for acute headaches attributed to ischemic stroke have been determined by the International Headache Society and are defined as (1) headaches that developed in close temporal relation to other symptoms and/or clinical signs of ischemic stroke or have led to the diagnosis of ischemic stroke, and (2) headaches that significantly improved in parallel with the stabilization or improvement of other symptoms or clinical or radiological signs of stroke [3]. Notably, headaches occur in 6–44% of ischemic stroke patients, as reported in two recent systematic reviews with meta-analyses [4,5]. Most patients experience headache symptoms on the day of stroke presentation or within the following days. Furthermore, given their high prevalence, post-stroke headaches can have a significant impact on recovery, quality of life, and even survival after a stroke. Leira et al. [6] showed that headache onset was a strong predictor of early neurological deterioration. In contrast, Chen et al. [7] argued that headache onset would be associated with modest but significantly better outcomes after ischemic stroke.

The most common types of post-stroke headaches are likely tension-type (50%) and migraine-like headaches (31%) [8]. While the pathophysiology of post-stroke headache has not yet been elucidated, various mechanisms have been suggested. Mechanical or chemical stimulation of trigeminovascular afferents innervating extra- and intracranial vessels or possible ischemia to the pain-sensitive dura and its stretch due to the massive cortical lesion may lead to sensitization of peripheral nociceptive pathways [9]. Sensitization, the enhanced responsiveness of peripheral and central neurons to repetitive nociceptive stimuli, can lead to hyperalgesia and allodynia providing the long-lasting time course of a (migraine-like) headache attack. In this scenario, the long-lasting character of migraine-like headaches might be supported by sensitization of the central nociceptive pathways after activation of the trigeminovascular system [10,11]. One of the specific symptoms of post-stroke migraine-like headaches is throbbing pain [3]. Our previous studies demonstrate the role of Piezo channels as mechanosensitive sensors in transcoding mechanical forces into electrical signals in trigeminal neurons, which contribute to the generation of throbbing pain [12,13]. Notably, activation of descending inhibitory projections to the brainstem neuronal network potentially diminishes the effectiveness of peripheral nociceptive traffic and prevents the involvement of central mechanisms of post-stroke headache [14]. Unlike hyperalgesia, which may be based on peripheral sensitization, cutaneous allodynia, the painful responsiveness to normally innocuous stimuli, is likely based on central mechanisms [15,16].

Apart from putative mechanosensitive ion channels, peripheral meningeal afferents are equipped with dual mechano- and chemosensitive transient receptor potential vanilloid 1 (TRPV1) channels [17] activated by endovanilloids [18] and endocannabinoids [19] or mechanical forces [20]. Likewise, mechanosensitivity of transient receptor potential cation channel subfamily M member 3 (TRPM3) channels in the meningeal afferents are highly sensitive to steroid hormones [13].

It is thus conceivable that mechanical pressure through tissue displacement and/or compression on pain-sensitive structures after severe ischemia activates mechanosensitive nociceptors such as Piezo1 channels [21,22]. Here we carried out a pilot study in mice to obtain the first insight into how headache pain may occur after experimental ischemic stroke. To this end, the activity of meningeal afferents was recorded in mice subjected to cortical photothrombosis (PT) and related to lesion size and behavioral impairment.

## 2. Results

### 2.1. Cortical Stroke after Photothrombosis

**Figure 1:**
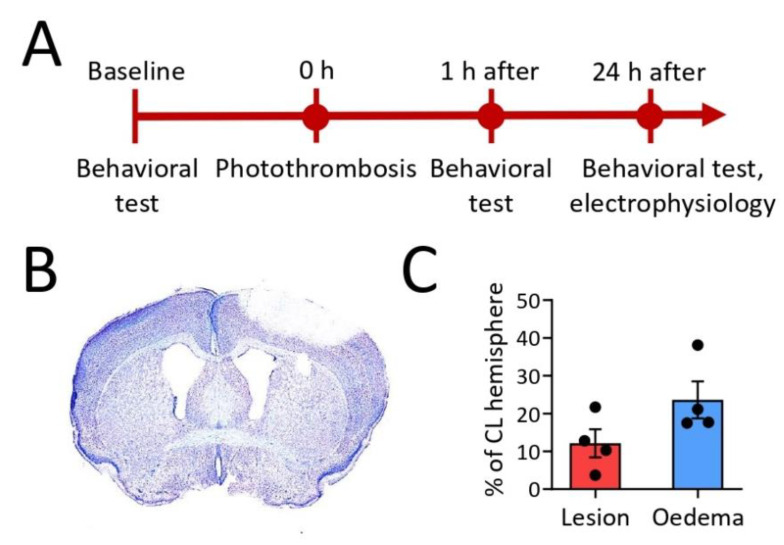
The study design (**A**). Typical ischemic damage to the motor cortex not affecting the corpus callosum in the ipsilateral (IL) hemisphere (**B**). Lesion size and edema as a percentage of the contralateral hemisphere (CL) (**C**).

Intravenous injection of light-sensitive Rose Bengal and cold light illumination over the right motor cortex produced a typical photothrombotic lesion. Figure 1B shows damage to the motor cortex, not affecting the corpus callosum. One of the five mice injected with Rose Bengal developed no lesion. At post hoc inspection, this mouse had signs of hydrocephalus, which can occasionally (0.03%) occur spontaneously in C57BL/6J mice. As a consequence, the mouse had considerably enlarged ventricles in both hemispheres and was excluded from further analysis. The lesion size for the remaining four mice, calculated as the percentage of the CL hemisphere, was 3.7–21.7% (Figure 1C). In the five control-operated mice, no lesions were observed. In the ischemic stroke group, variable edema formation was observed in the IL hemisphere (17.5–38.1%) (Figure 1C).

### 2.2. Motor Function and Mechanical Allodynia Sensitivity after Cortical Photothrombosis

**Figure 2:**
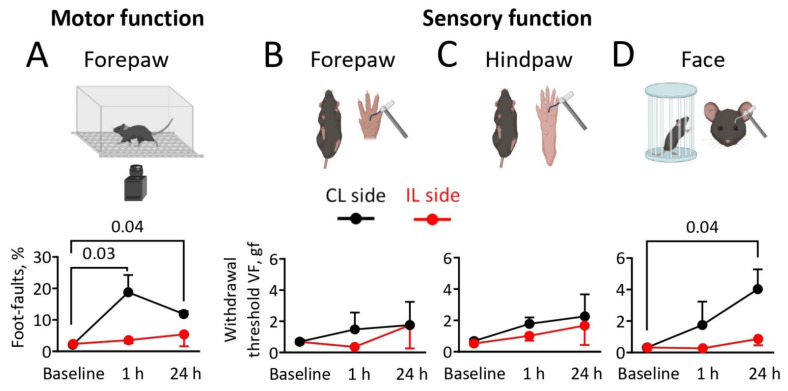
Behavioral assessment of sensorimotor functions. The grid-walking test was used to measure motor functions. The graph shows the foot-fault ratio (foot-faults vs. total steps) for ipsilateral (IL) (red) and contralateral (CL) (black) forepaws during baseline and after 1 and 24 h of cortical photothrombosis (PT). Notice the increased percentage of foot-faults after 1 (*p* = 0.03) and 24 h (*p* = 0.04) on the CL forepaws in comparison to baseline motor activity (**A**). Schematic representation of a sensory function in ischemic stroke mice forepaws using von Frey filaments. The graph shows the maximum g force (gf) of von Frey filaments used as a withdrawal threshold for IL (red) and CL (black) forepaws during baseline, after 1 and 24 h of PT (**B**). Sensory functions of hindpaws were tested in the same manner using von Frey filaments (**C**). Schematic representation of testing setup for sensory functions of periorbital face area using von Frey filaments. Of note, the von Frey filaments gf was increased for the CL periorbital face area after 24 h of PT in comparison to baseline (*p* = 0.04) (**D**). Data are mean ± SEM. Figure was created with BioRender.com. (accessed on 16 March 2022).

Next, we evaluated to what extent PT affected sensorimotor functions. The grid-walking test was used to assess motor function and coordination. At the acute 1-h time point, FF of the CL forepaws was increased (18.7 ± 5.5%, *p* = 0.03) and remained high at the 24-h time point (11.8 ± 0.9%, *p* = 0.04) compared to the baseline (2.0 ± 0.4%), indicating a severe motor impairment after ischemic stroke (Figure 2A). The function of the IL forepaws was not affected. FF of the hindpaws was too rare to be analyzed.

We also assessed the mechanical pain sensation threshold in the mice subjected to ischemic stroke. To this end, we performed a test for mechanical allodynia using von Frey filaments from 0.26 to 6.2 gf. von Frey testing showed that the sensitivity to mechanical allodynia for CL or IL forepaws and hindpaws in mice was not different at the 1-h and 24-h time points compared to baseline (Figure 2B,C). Instead, the threshold for CL face mechanical allodynia sensitivity increased at the 24-h time point after PT compared to the baseline (*p* = 0.04, Figure 2D). Face mechanical allodynia sensitivity of the IL side was not affected.

In conclusion, cortical PT produced a variable-sized lesion with edema, immediate impairment of motor function, and a steady decline of sensitivity to mechanical stimuli in the CL side of the face.

### 2.3. Electrophysiological Observations in Meningeal Nerves in Relation to Lesion and Edema Formation

**Figure 3:**
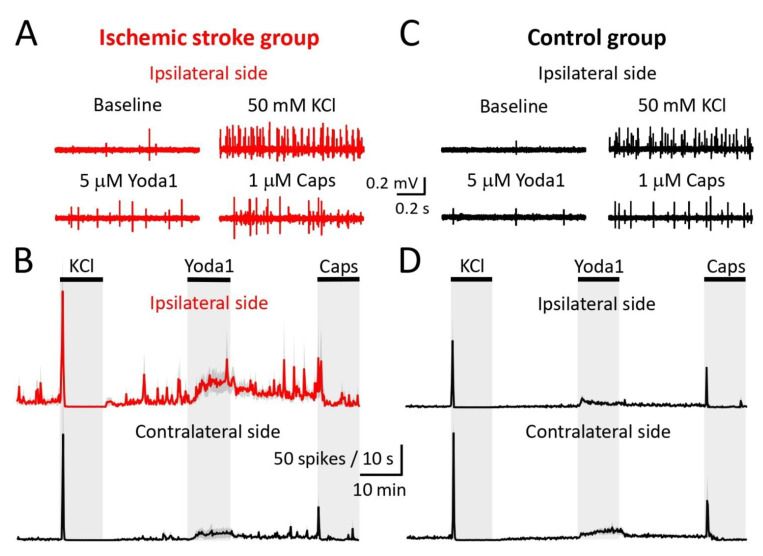
Examples of original electrophysiological recordings for the ipsilateral (IL) hemi-skull of the ischemic stroke group at baseline and during 50 mM KCl, 5 μM Yoda1, and 1 μM capsaicin applications (**A**). Time course of spike frequency (10 s bin size) induced by application of 50 mM KCl, 5 μM Yoda1, and 1 μM capsaicin, presented for both IL and contralateral (CL) hemi-skulls of the ischemic stroke group (**B**). Examples of original electrophysiological recordings for the IL hemi-skull of the control group at baseline and during 50 mM KCl, 5 μM Yoda1, and 1 μM capsaicin applications (**C**). Time course of spike frequency (10 s bin size) induced by application of 50 mM KCl, 5 μM Yoda1, and 1 μM capsaicin, presented for both IL and Cl hemi-skulls of the control group. Of note, high-frequency fluctuations occur in the IL hemi-skulls of the ischemic stroke group throughout the recording period (**D**). Data are mean ± SEM.

**Figure 4:**
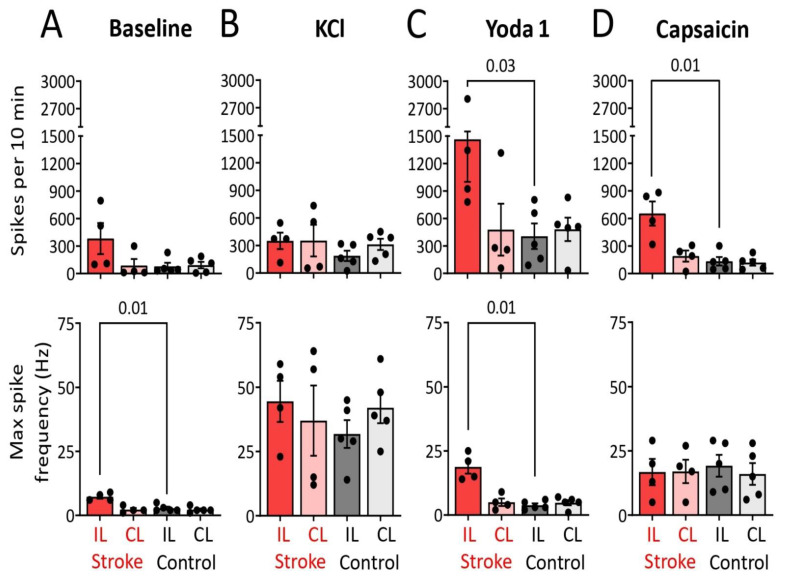
Histograms (top) depict the number of nociceptive spikes during 10 min recording (baseline) for the ipsilateral (IL) and contralateral (CL) hemi-skulls in the ischemic stroke and control groups. Histograms (bottom) depict the mean number of maximal spike frequency at baseline for the IL and CL hemi-skulls in the ischemic stroke and control groups. Notice the maximal spike frequency is higher in the IL hemi-skulls of the ischemic stroke group compared to those in the control group (*p* = 0.01) (**A**). Histograms (top) depict the number of nociceptive spikes during 10 min recordings after 50 mM KCL application for the IL and CL hemi-skulls in the ischemic stroke and control groups. Histograms (bottom) depict the number of maximal spike frequency during 50 mM KCL for the IL and CL hemi-skulls in ischemic stroke and control groups (**B**). Histograms (top) depict the number of nociceptive spikes during 10 min recordings after application of 5 μM Yoda1 for the IL and CL hemi-skulls in the ischemic stroke and control groups. Of note, firing is increased in the IL hemi-skulls of the ischemic stroke group compared to the corresponding hemi-skulls of the control group (*p* = 0.03). Histograms (bottom) depict the number of maximal spike frequency during application of 5 μM Yoda1 for the IL and CL hemi-skulls in the ischemic stroke and control groups, where maximal spike frequency is higher in the IL hemi-skulls of the ischemic stroke group compared to those in the control group (*p* = 0.01) (**C**). Histograms (top) depict the number of nociceptive spikes during 10 min recordings after application of 1 μM capsaicin for the IL and CL hemi-skulls in the ischemic stroke and control groups. Notice that in the IL hemi-skulls of the ischemic stroke group, capsaicin induces nociceptive firing continuously throughout entire 10 min, increasing the number of spikes compared to the IL hemi-skulls of the control group (*p* = 0.01). The bottom histograms depict the mean number of maximal spike frequency during 1 μM capsaicin application for the IL and CL hemi-skulls in the ischemic stroke and control groups (**D**). Data are mean ± SEM.

Electrophysiological measurements of nociceptive firing were recorded from meningeal afferents of hemi-skull preparations as it has relevance to understanding post-stroke headache pain. Figure 3 shows examples of original electrophysiological recordings and spike time courses, gained from hemi-skulls from mice subjected to cortical PT (Figure 3A,B), or cold light illumination without Rose Bengal infusion (Figure 3C,D). Examples of gained recordings are presented only for the IL hemi-skull, whereas time courses are presented separately for IL and CL hemi-skulls. Statistical analysis revealed that spiking activity in meningeal nerve afferents of the IL hemi-skulls with a lesion was not significantly higher at baseline, i.e., before any compound was applied, compared to the CL hemi-skull of the same mouse or when compared to IL and CL hemi-skulls of the control-operated mice (Figure 4A (top)). However, by calculating maximal spike frequency, we found that at baseline, the trigeminal meningeal afferents in the IL hemi-skull of the ischemic stroke group were more capable of higher-frequency spiking activity compared to meningeal afferents in the IL hemi-skull of the control mice (7.3 ± 0.7 vs. 2.8 ± 0.6, *p* = 0.01, Figure 4A (bottom)). During the 10 min activation of mechanosensitive Piezo1 channels with the specific agonist Yoda1 (5 μM), spiking activity was higher in the IL hemi-skulls of the mice subjected to ischemic stroke compared to the IL hemi-skulls of the control-operated mice (1464 ± 463.5 vs. 405.6 ± 139.7, *p* = 0.03, Figure 4C (top)). Moreover, during the Yoda1 application, the maximal spike frequency remained higher in the IL hemi-skulls of mice of the ischemic stroke group compared to those of the control group (18.8 ± 2.6 vs. 3.8 ± 0.7, *p* = 0.01, Figure 4C (bottom)). TRPV1 channels were continuously active during the entire 10 min of capsaicin (1 µM) application in the IL hemi-skulls of the ischemic stroke group, increasing the number of spikes compared to IL hemi-skulls of the control group where TRPV1 channels were active for 30 s (653.5 ± 131 vs. 133.2 ± 46, *p* = 0.01, Figure 4D (top)). Although, inside this active 30 s phase, the maximal spike frequency of TRPV1 channel induction by capsaicin (1 µM) did not differ across all groups (Figure 4D (bottom)). The fact that general excitability induced by 50 mM KCl and measured by the sum of spikes per 10 min (Figure 4B (top)) and the maximal spike frequency (Figure 4B (bottom)) was equal across groups demonstrates that the experiments were technically performed under similar conditions.

## 3. Discussion

The present study is the first to shed light on whether ischemic stroke activates peripheral meningeal afferents, a mechanism known to generate head pain [23]. We used the photothrombotic ischemic stroke model, which causes peroxidative damage of the endothelial membrane, platelet adhesion and aggregation, and eventually thrombus formation in microcapillaries and thereby an ischemic lesion [24,25,26]. Our data revealed enhanced spiking activity after activation of mechanosensitive Piezo1 channels in trigeminal afferents of the IL hemi-skulls of the ischemic stroke group, as well as prolonged activation of nociceptive TRPV1 channels. Interestingly, the frequency of spiking activity was also higher in these meningeal afferents at baseline and during the activation of mechanosensitive Piezo1 channels. Moreover, these phenomena were shown to be associated with impaired sensorimotor function. Perhaps surprisingly at first glance, the threshold for contralateral face mechanical allodynia sensitivity was increased, but this is possibly due to damage to the sensorimotor cortex. Taken together, our data suggest that cortical stroke increases IL nociceptive firing from trigeminal afferents that are possibly involved in post-stroke headaches.

Various experimental models, including the most commonly used transient (tMCAO) and permanent MCAO (pMCAO), optogenetically triggered cortical spreading depolarization (CSD), and i.v. microsphere embolization are used to produce stroke-like events in animals [27,28,29]. Notably, most methods cause considerable trauma to the animal due to craniotomy and/or the manipulation of internal or external carotid arteries with surgical procedures. In addition, MCAO and thromboembolic models are associated with subarachnoid hemorrhage and blocking of the hypothalamic blood supply leading to hypothalamic infarction and, subsequently, hyperthermia that worsens outcomes [30]. Concerning i.v. microspheres, the major problem is variable locations of multiple small lesions without a robust behavioral deficit. Whereas optogenetically triggered CSD has been linked to stroke pathology, only a transient neurobehavioral deficit is observed [31]. Therefore, for our study, we used the minimally invasive cortical PT. When cold light is used, a penumbral region is present with a decrease in blood flow and relevant stroke pathology, including inflammation and altered excitability. In this model, lesion location and size can be adjusted and controlled at a distance far from the site of the electrophysiological recording. The choice of lesion coordinates was based on Clarkson et al. [32] by which we targeted both the forelimb/hindlimb motor area. In our hands, the lesion in the motor cortex produces a long-lasting sensorimotor impairment [33]. Moreover, photothrombosis is recommended particularly for long-term functional recovery studies over tMCAO (filament) and pMCAO models [34].

The primary behavioral readout in our study was the grid-walking test, which showed a robust acute motor impairment of the CL forelimb. As it is challenging to differentiate between acute stroke impairment and headache/migraine-like behavior in an animal study, we mainly focused on identifying possible changes in mechanical allodynia sensitivity in the area of trigeminal nerve innervation. It is known that the forelimb area of the motor cortex overlaps with the primary somatosensory region in rodents and that the motor cortex is important in the processing of somatosensation via its anatomical and functional connections. Thus, the von Frey test in our study more likely indicates ischemic stroke-related damage to the sensorimotor cortex, which we observed by the steady decline in sensory function on the CL side starting from 1 h and reaching a difference with baseline at the 24 h time point.

The electrophysiological data were expected to advance our understanding of the mechanisms of headache generation after stroke. The isolated hemi-skull preparation is regarded as a reliable model for studying peripheral neuronal mechanisms [35,36]. In addition, the preservation of the basic morphology of meningeal tissue including the meningeal nerves is an advantage of this *ex vivo* preparation. The main meningeal branch used for our recordings mainly innervates the region of the middle meningeal artery (MMA), known to be a key contributor to various types of headaches [37]. Moreover, our approach allowed us to record exclusively peripheral nociceptive traffic, excluding spikes that could originate from the somas of neurons in the trigeminal ganglion [38,39]. We recorded the absolute number of spontaneous (baseline) and evoked spikes by Yoda1 and capsaicin for 10 min each from trigeminal nerve terminals that are deemed relevant specifically for migraine-like headaches [17,39,40,41]. The difference between the sum of spikes in response to the various agonists across different groups provides meaningful insight into the impact of ischemic stroke on the peripheral area of the trigeminovascular system. Furthermore, as the intensity of the pain depends not only on the total number of spikes generated in the nociceptive fibers but also on the frequency of spiking activity [42,43], we compared the maximal spike frequencies at baseline and for all agonists across groups.

Our main observation is the generation of high-frequency spikes, albeit with considerable fluctuations throughout the recording in the IL hemi-skull. Such spiking activity generated from peripheral meningeal nerve fibers that are part of the trigeminovascular system can be interpreted as an intracranial physiological manifestation of stroke headaches. In line with our experimental data, observational studies of patients with acute ischemic stroke report, in 15 to 40% of the cases, an IL headache occurring in close temporal relation to the ischemic event [44]. The pathophysiology of post-stroke headache has been suggested to include edema formation, hemorrhagic transformation, and indeed changes in the trigeminovascular system [43]. Consistent with this notion, we not only observed the lesion but also an increased firing of intracranial meningeal nerve fibers in the same hemi-skull at the acute stage of stroke.

In previous studies, we established functional expression of mechanosensitive Piezo1 channels in peripheral meningeal nerve fibers [12,45,46]. Yoda1 was discovered after screening several million compounds and validated as a selective Piezo1 agonist not acting on related Piezo2 channels [47]. Additionally, Yoda1 specificity was also confirmed in the rodent trigeminal neurons by blocking its activation with the selective Piezo1 antagonist Dooku [44]. Notably, activation of these channels is considered a mechanism involved in typical headache symptoms, such as mechanical hyperalgesia [12,13,46]. Piezo1 channels are activated by mechanical force [48] which may come from pulsating and dilating vasculature due to the release of the neuropeptide calcitonin gene-related peptide (CGRP) from intracranial vessels [46]. We show that the sensitivity of these channels occurred exclusively in the hemi-skulls of the hemisphere in which the lesion had occurred. We also observe that only on these hemi-skulls do meningeal afferents have more ability to transmit high-frequency spiking activity, which indicates that this site is involved more in the generation of pain [49]. Thus, the ischemic lesion, together with pulsating vessels, acts as mechanical triggers to activate these mechanosensitive channels in meningeal afferents. This scenario seems relevant to clinical observations where the ischemic stroke is accompanied by cerebral edema and swollen tissues in a fixed volume of the skull, exerting a mechanical force on adjacent tissues and capillaries leading to decreased blood perfusion, aggravated ischemia and edema, and tissue damage [50,51,52,53]. Consistent with this view, a recent observation has shown that the activity of Piezo1 channels correlated with the severity of brain edema in glioblastomas and that up-regulation of the expression of Piezo1 channels correlated to the size of brain edema [54]. It has also been shown that the expression of Piezo1 channels is increased after cerebral ischemia in rats [55]. Moreover, the inhibition of Piezo1 channels effectively inhibited arterial thrombosis and reduced the infarct size in animal stroke models [56]. Our observation that TRPV1 channels were activated for the entire 10 min period in the ischemic stroke group indicates that these channels are also involved in post-stroke headache. Interestingly, the involvement of TRPV1 channels in stroke pathophysiology was also shown in other studies [57,58]. We suggest that the release of inflammatory mediators and their penetration through already damaged blood-brain barrier (BBB) beneath the meninges may stimulate TRPV1 channels on the trigeminal nerve terminals, contributing to the generation of nociceptive signaling. Consistent with this, it has been shown that reduced activity of TRPV1 channels alleviates the inflammatory response induced by ischemic damage [59].

Notably, polymodal TRPV1 channels, apart from chemical agonists capsaicin, endogenous vanilloids, and endocannabinoids [18,19], can also be activated by mechanical force [20]. Thus, distinct stimuli can provide a synergic effect in pain signaling via TRPV1 receptors. Moreover, the co-activation of Piezo1 and TRPV1 nociceptive channels after stroke raises the interesting issue of potential functional interactions between these two signaling pathways, likely implicated in migraines. That is, the simultaneous activation of both receptors could provide a supra-additive pro-nociceptive effect, analogous to functional interactions between NMDA and TRPV1 receptors [60]. In contrast, when expressed in the same nerve fiber, previous extensive activation of TRPV1 channels associated with massive calcium influx can potentially result in PLC/lipid signaling-mediated reduced Piezo1 activity [61].

Early peripheral activation of the trigeminal system can further engage progressive sensitization of the central nociceptive pathways, supporting the long-lasting character of migraine-like headaches [10,11]. However, the activation of descending inhibitory projections to the brainstem neuronal network can diminish the effectiveness of the peripheral nociceptive traffic and prevent the involvement of central mechanisms of migraine-like headaches [14]. It has been proposed that ‘descending analgesic systems seem “switched off” in generalized allodynia‘ [16]. However, in our case, ischemic damage to the motor cortex likely also affects cortical areas implicated in processing nociceptive signaling.

The small sample size is the major limitation of this study. Therefore, we have framed the work as an exploratory, pilot study generating new hypotheses and pathophysiological theories of disease. By this, we mean that exploratory studies capture something broader than what is generally customary in statistics [62]. Given the robust effect in electrophysiology, future studies with a larger sample size using additional stroke models are needed to confirm the role of meningeal afferents in trigeminal headaches after a stroke. In addition, long-term follow-up studies using a battery of sensitive tests for both sensorimotor impairment and pain may shed new light on the chronic phase of post-stroke headache.

In summary, we propose that in stroke-related conditions, mechanosensitive Piezo1 channels and nociceptive TRPV1 channels in meningeal afferents are sensitized and thus may contribute to the generation of post-stroke headaches. As the activation of these channels in meningeal afferents has previously been proposed to play a role in migraines [12,13,39], it is tempting to speculate that mechanisms converging on Piezo1 and TRPV1 channels play a role in various types of headaches.

## 4. Materials and Methods

### 4.1. Animals

Male C57BL/6J mice (Jackson Laboratory, Bar Harbor, ME, USA) at 8–12 weeks of age were used. Mice were housed in the Animal Facility of the University of Eastern Finland in IVC cages with a controlled temperature of 22 °C, humidity, and a 12-h light/12-h dark cycle with food and water ad libitum. All animal procedures were approved by the Animal Ethics Committee (Hämeenlinna, Finland), and conducted in accordance with the guidelines set by the European Community Council Directives 86/609/EEC. All efforts were made to minimize the number of animals used and their suffering.

### 4.2. Cortical Photothrombosis

To induce cortical photothrombosis, Rose Bengal (Sigma, Ronkonkoma, NY, USA) was dissolved in 0.9% NaCl to a final concentration of 15 mg/mL, filtered (Chromafil Xtra, pore size 1.2 μm), and injected into the tail vein (3.3 µL/g) of five mice (Ischemic stroke group) (Figure 1A). Subsequently, mice were anesthetized with isoflurane (5% for induction, 2% for maintenance) and mounted onto a stereotactic frame (David Kopf Instruments, Tujunga, CA, USA). The skull was exposed with a ~1-cm incision along the midline. A cold light source (150 W; Highlight 2100, Olympus Europe, Hamburg, Germany) was placed on the skull over the right motor cortex at the following stereotaxic coordinates: AP: +1.1 mm from Bregma; L: + 2.0 mm [33]. The light source with a 3-mm aperture was switched on for 10 min. Released free radicals cause endothelial damage and the formation of a thrombus in microcapillaries and a subsequent ischemic stroke [24,25]. After surgery, the skin was sutured and the mice were treated for post-operative pain with Norocarp Vet (50 mg/kg, s.c., Norbrook Laboratories Limited, Newry, Northern Ireland). Five control mice (Control group) underwent the same procedure with cold light exposure albeit without Rose Bengal injection.

### 4.3. Behavioral Testing

Mice were subjected to behavioral testing, which was carried out before PT (baseline), as well as 1 h (acute testing) and 24 h thereafter. The grid-walking test was used to measure limb placement deficits and motor coordination during locomotion. The test apparatus was made using a 12-mm square wire mesh with a grid area of 15 cm (length) × 20 cm (width). A video camera was placed beneath the grid to allow the recording of foot faults (FF) during a period of 3 min. The number of FF for each forelimb along with the number of non-FF steps were counted and a ratio was calculated between FF and the total steps taken [32].

After the grid-walking test, von Frey mechanical allodynia testing of the paws was performed. Mice were kept on the walking grid to access both forepaws and hindpaws from underneath the mesh that was covered with a 5 cm × 5 cm box to restrict animal movement. Before testing, mice were allowed to habituate in the testing chamber for 10 min. Calibrated von Frey filaments were placed perpendicularly to the glabrous skin at the midplantar surface of both forepaws and hindpaws, while avoiding touching toes. Applications were started from the filament strength, 0.26 g force (gf), and with force just enough to slightly bend the filament against the paw. Four stimuli with one filament were applied with the interval between the stimuli being 4–5 seconds. If there were not four positive responses (100%), the next filament with a higher gr was taken after a 30-s waiting period to avoid wind-up effect. The process was repeated until a filament reached a 100% response or until the filament with the highest (6.2) gf was applied. Flinching or sudden paw withdrawal was considered a positive response, while no response, toe flaring, or ambulation was considered a negative response. The gf of the last von Frey filament applied to reach a 100% response (withdrawal threshold) was used for further analysis.

For von Frey’s mechanical allodynia testing of the head, a cylindrical wire mesh was used to allow access to the periorbital face area. Mice were allowed to habituate for 10 min before testing began. As with the paws, von Frey filaments were applied perpendicular to the left and right periorbital area between the caudal portion of the eyes and the ear’s tragus, while paying close attention to avoid touching whiskers or the surgery area. Four stimuli were applied at an interval of 4–5 s until four subsequent responses were obtained. If head escape behavior was observed at least four times, the mouse was considered to be responsive to that filament, and the force of that filament was recorded. Positive responses were defined if an animal slowly turned the head away or briskly moved the head backwards, spontaneously and rapidly shook its head, and actively attacked the stimulus object making biting and grabbing movements. If there was no response for one of the four applications, the next filament with a higher gf was used after a 30-s waiting period.

### 4.4. Tissue Preparation

Mice were sacrificed 24 h after the operation by cervical dislocation. Brains were carefully removed from the skull, post-fixed overnight in 4% paraformaldehyde in 0.1 M phosphate buffer (pH 7.4), and cryoprotected in 30% sucrose. The brains were cut into sections (35 μm) using a sliding microtome (Leica, Wetzlar, Germany), and sections were stored in an anti-freeze solution at −20 °C until use. For each mouse, 10 sections through the lesion were picked and Nissl-stained for measurement of infarct size [63] and edema [64].

### 4.5. Electrophysiology

Both ipsilateral (IL) and contralateral (CL) hemi-skulls were used for electrical recordings of spikes. To record nociceptive firing from the peripheral part of trigeminal nerves innervating the meninges, direct electrophysiological spike recording from hemi-skull preparations was performed for both the ischemic stroke and control groups, using an approach described previously [17]. Briefly, immediately after animal sacrificing, hemi-skull preparations were cleaned, keeping the meninges untouched, and placed in a recording bath with continuous perfusion by oxygenated artificial cerebrospinal fluid (aCSF) containing 120 mM NaCl, 2.5 mM KCl, 2 mM CaCl_2_, 1 mM MgCl_2_, 11 mM glucose, 24 mM NaHPO_4_, and 30 mM NaHCO_3_, bubbled with 95% O_2_/5% CO_2_ at room temperature (RT) with pH maintained at 7.25–7.35 and constant flow of liquid (6–7 mL/min). Next, using a 30 G needle, at a distance of ~0.5 mm from the trigeminal ganglion, a small incision was made in the dura mater at both sides of the meningeal nerve, which was then cut. The distal part of the cut meningeal nerve was sucked inside a glass recording microelectrode that was filled with aCSF.

To stabilize the baseline and assess a difference in basal activity between groups, 20 min of spontaneous spiking activity was recorded at the start of each experiment. After that, to test general nerve excitability, first, 50 mM KCl with compensated osmolarity was applied. Next, to study the activity of mechanosensitive Piezo1 channels, the specific agonist Yoda1 (5 μM, Tocris Bioscience, Bristol, UK) was applied. At the end of each experiment, to assess the output of nociceptive neuronal activity from TRPV1 receptors, capsaicin (1 μM, Tocris Bioscience, UK) was applied. Drugs were dissolved in DMSO and diluted to a final concentration in aCSF immediately before usage. The same concentration of DMSO was administered before compound application, which did not influence the nociceptive firing of the meningeal nerve. All drugs were administrated to the same receptive field around the main meningeal nerve, and the application lasted 10 min with subsequent washout for 20 min.

Recordings of spontaneous and stimulated activity generated in the peripheral part of the meningeal nerves were conducted at RT using a low-noise digital amplifier (ISO-80; World Precision Instruments, Sarasota, FL, USA) with gain of 10,000× and bandpass of 300–3000 Hz. Obtained signals were digitized at 8-µsec intervals using a NIPCI 6221 data acquisition board (National Instruments, Austin, TX, USA) and stored on a PC for further offline analysis. Electrical signals were visualized with WinEDR v.3.5.2 software (University of Strathclyde, Glasgow, UK) and analyzed with MATLAB-based software (MathWorks, Natick, MA, USA). Results are presented as the sum of the original numbers of spikes per experiment for all agonists during the 10 min of recording (baseline, Yoda1, KCl and capsaicin). Additionally, the maximal spike frequency (in Hz) at baseline and in response to the various agonists was calculated. Electrophysiological data were plotted using GraphPad Prism (GraphPad Prism Software, San Diego, CA, USA).

### 4.6. Statistics

Statistical analyses for morphology, behavioral, and electrophysiological data were performed using SPSS software for Windows (version 27). Based on a power analysis (*α* = 0.05, *β* = 0.8) of electrophysiological data from the group, we tested 5 mice per group. The Mann–Whitney U test was used to test for differences between the ischemic stroke and control groups. The Wilcoxon rank-sum test was used to assess differences between the IL and the CL side. The Friedman test was used to assess differences in behavior between the three time points (baseline, 1, and 24 h). Results are expressed as the mean ± standard error of the mean (SEM) or as the median with the interquartile range.

## Data Availability

The datasets generated and analyzed for this are available from the corresponding author upon reasonable request.

## References

[1] Wilkins E., Wilson L., Wickramasinghe K., Bhatnagar P., Leal J., Luengo-Fernandez R., Burns R., Rayner M., Townsend N. European Cardiovascular Disease Statistics 2017. http://www.ehnheart.org/images/CVD-statistics-report-August-2017.pdf.

[2] Balami J.S., Chen R.-L., Grunwald I.Q., Buchan A.M. (2011). Neurological Complications of Acute Ischaemic Stroke. Lancet Neurol..

[3] (2018). Headache Classification Committee of the International Headache Society (IHS) The International Classification of Headache Disorders, 3rd Edition. Cephalalgia.

[4] Oliveira F.A.A., Sampaio Rocha-Filho P.A. (2019). Headaches Attributed to Ischemic Stroke and Transient Ischemic Attack. Headache.

[5] Harriott A.M., Karakaya F., Ayata C. (2020). Headache after Ischemic Stroke: A Systematic Review and Meta-Analysis. Neurology.

[6] Leira R., Dávalos A., Aneiros A., Serena J., Pumar J.M., Castillo J. (2002). Headache as a Surrogate Marker of the Molecular Mechanisms Implicated in Progressing Stroke. Cephalalgia.

[7] Chen P.-K., Chiu P.-Y., Tsai I.-J., Tseng H.-P., Chen J.-R., Yeh S.-J., Yeh S.-J., Sheu J.-J., Chung C.-P., Wu M.-H. (2013). Onset Headache Predicts Good Outcome in Patients with First-Ever Ischemic Stroke. Stroke.

[8] Hansen A.P., Marcussen N.S., Klit H., Kasch H., Jensen T.S., Finnerup N.B. (2015). Development of Persistent Headache Following Stroke: A 3-Year Follow-Up. Cephalalgia.

[9] Evans R.W., Mitsias P.D. (2009). Headache at Onset of Acute Cerebral Ischemia. Headache.

[10] Pietrobon D., Moskowitz M.A. (2013). Pathophysiology of Migraine. Annu. Rev. Physiol..

[11] Noseda R., Burstein R. (2013). Migraine Pathophysiology: Anatomy of the Trigeminovascular Pathway and Associated Neurological Symptoms, Cortical Spreading Depression, Sensitization, and Modulation of Pain. Pain.

[12] Mikhailov N., Leskinen J., Fagerlund I., Poguzhelskaya E., Giniatullina R., Gafurov O., Malm T., Karjalainen T., Gröhn O., Giniatullin R. (2019). Mechanosensitive Meningeal Nociception via Piezo Channels: Implications for Pulsatile Pain in Migraine?. Neuropharmacology.

[13] Krivoshein G., Tolner E.A., van den Maagdenberg A., Giniatullin R.A. (2022). Migraine-Relevant Sex-Dependent Activation of Mouse Meningeal Afferents by TRPM3 Agonists. J. Headache Pain.

[14] Giniatullin R. (2022). 5-Hydroxytryptamine in Migraine: The Puzzling Role of Ionotropic 5-HT3 Receptor in the Context of Established Therapeutic Effect of Metabotropic 5-HT1 Subtypes. Br. J. Pharmacol..

[15] Russo A., Esposito F., Conte F., Fratello M., Caiazzo G., Marcuccio L., Giordano A., Tedeschi G., Tessitore A. (2017). Functional Interictal Changes of Pain Processing in Migraine with Ictal Cutaneous Allodynia. Cephalalgia.

[16] Maleki N., Szabo E., Becerra L., Moulton E., Scrivani S.J., Burstein R., Borsook D. (2021). Ictal and Interictal Brain Activation in Episodic Migraine: Neural Basis for Extent of Allodynia. PLoS ONE.

[17] Zakharov A., Vitale C., Kilinc E., Koroleva K., Fayuk D., Shelukhina I., Naumenko N., Skorinkin A., Khazipov R., Giniatullin R. (2015). Hunting for Origins of Migraine Pain: Cluster Analysis of Spontaneous and Capsaicin-Induced Firing in Meningeal Trigeminal Nerve Fibers. Front Cell Neurosci..

[18] Dux M., Deák É., Tassi N., Sántha P., Jancsó G. (2016). Endovanilloids Are Potential Activators of the Trigeminovascular Nocisensor Complex. J. Headache Pain.

[19] Della Pietra A., Savinainen J., Giniatullin R. (2022). Inhibiting Endocannabinoid Hydrolysis as Emerging Analgesic Strategy Targeting a Spectrum of Ion Channels Implicated in Migraine Pain. Int. J. Mol. Sci..

[20] Stewart L., Turner N.A. (2021). Channelling the Force to Reprogram the Matrix: Mechanosensitive Ion Channels in Cardiac Fibroblasts. Cells.

[21] Wang J., La J.-H., Hamill O.P. (2019). PIEZO1 Is Selectively Expressed in Small Diameter Mouse DRG Neurons Distinct from Neurons Strongly Expressing TRPV1. Front Mol. Neurosci..

[22] Guo X.-W., Lu Y., Zhang H., Huang J.-Q., Li Y.-W. (2021). PIEZO1 Might Be Involved in Cerebral Ischemia-Reperfusion Injury through Ferroptosis Regulation: A Hypothesis. Med. Hypotheses.

[23] Moskowitz M.A. (1993). Neurogenic Inflammation in the Pathophysiology and Treatment of Migraine. Neurology.

[24] Watson B.D., Dietrich W.D., Busto R., Wachtel M.S., Ginsberg M.D. (1985). Induction of Reproducible Brain Infarction by Photochemically Initiated Thrombosis. Ann. Neurol..

[25] Lipsanen A., Flunkert S., Kuptsova K., Hiltunen M., Windisch M., Hutter-Paier B., Jolkkonen J. (2013). Non-Selective Calcium Channel Blocker Bepridil Decreases Secondary Pathology in Mice after Photothrombotic Cortical Lesion. PLoS ONE.

[26] Orset C., Macrez R., Young A.R., Panthou D., Angles-Cano E., Maubert E., Agin V., Vivien D. (2007). Mouse Model of in Situ Thromboembolic Stroke and Reperfusion. Stroke.

[27] Lipsanen A., Jolkkonen J. (2011). Experimental Approaches to Study Functional Recovery Following Cerebral Ischemia. Cell. Mol. Life Sci..

[28] Durukan A., Tatlisumak T. (2007). Acute Ischemic Stroke: Overview of Major Experimental Rodent Models, Pathophysiology, and Therapy of Focal Cerebral Ischemia. Pharmacol. Biochem. Behav..

[29] Kumar A., Aakriti, Gupta V. (2016). A Review on Animal Models of Stroke: An Update. Brain Res. Bull..

[30] Li F., Omae T., Fisher M. (1999). Spontaneous Hyperthermia and Its Mechanism in the Intraluminal Suture Middle Cerebral Artery Occlusion Model of Rats. Stroke.

[31] Alexis N.E., Back T., Zhao W., Dietrich W.D., Watson B.D., Ginsberg M.D. (1996). Neurobehavioral Consequences of Induced Spreading Depression Following Photothrombotic Middle Cerebral Artery Occlusion. Brain Res..

[32] Clarkson A.N., Huang B.S., Macisaac S.E., Mody I., Carmichael S.T. (2010). Reducing Excessive GABA-Mediated Tonic Inhibition Promotes Functional Recovery after Stroke. Nature.

[33] Välimäki N.-N., Bakreen A., Häkli S., Dhungana H., Keuters M.H., Dunlop Y., Koskuvi M., Keksa-Goldsteine V., Oksanen M., Jäntti H. (2022). Astrocyte Progenitors Derived from Patients With Alzheimer Disease Do Not Impair Stroke Recovery in Mice. Stroke.

[34] Corbett D., Carmichael S.T., Murphy T.H., Jones T.A., Schwab M.E., Jolkkonen J., Clarkson A.N., Dancause N., Wieloch T., Johansen-Berg H. (2017). Enhancing the Alignment of the Preclinical and Clinical Stroke Recovery Research Pipeline: Consensus-Based Core Recommendations from the Stroke Recovery and Rehabilitation Roundtable Translational Working Group. Int. J. Stroke.

[35] De Col R., Messlinger K., Carr R.W. (2012). Repetitive Activity Slows Axonal Conduction Velocity and Concomitantly Increases Mechanical Activation Threshold in Single Axons of the Rat Cranial Dura. J. Physiol..

[36] Uebner M., Carr R.W., Messlinger K., De Col R. (2014). Activity-Dependent Sensory Signal Processing in Mechanically Responsive Slowly Conducting Meningeal Afferents. J. Neurophysiol..

[37] Amir R., Devor M. (1996). Chemically Mediated Cross-Excitation in Rat Dorsal Root Ganglia. J. Neurosci..

[38] Thalakoti S., Patil V.V., Damodaram S., Vause C.V., Langford L.E., Freeman S.E., Durham P.L. (2007). Neuron-Glia Signaling in Trigeminal Ganglion: Implications for Migraine Pathology. Headache.

[39] Goadsby P.J. (2007). Recent Advances in Understanding Migraine Mechanisms, Molecules and Therapeutics. Trends Mol. Med..

[40] Levy D. (2010). Migraine Pain and Nociceptor Activation--Where Do We Stand?. Headache.

[41] Messlinger K. (2009). Migraine: Where and How Does the Pain Originate?. Exp. Brain Res..

[42] Tong C.-K., MacDermott A.B. (2014). Synaptic GluN2A and GluN2B Containing NMDA Receptors within the Superficial Dorsal Horn Activated Following Primary Afferent Stimulation. J. Neurosci..

[43] Zhang X.-F., Zhu C.Z., Thimmapaya R., Choi W.S., Honore P., Scott V.E., Kroeger P.E., Sullivan J.P., Faltynek C.R., Gopalakrishnan M. (2004). Differential Action Potentials and Firing Patterns in Injured and Uninjured Small Dorsal Root Ganglion Neurons after Nerve Injury. Brain Res..

[44] Diener H.C., Katsarava Z., Weimar C. (2008). Headache Associated with Ischemic Cerebrovascular Disease. Rev. Neurol..

[45] Mikhailov N., Plotnikova L., Singh P., Giniatullin R., Hämäläinen R.H. (2022). Functional Characterization of Mechanosensitive Piezo1 Channels in Trigeminal and Somatic Nerves in a Neuron-on-Chip Model. Int. J. Mol. Sci..

[46] Della Pietra A., Mikhailov N., Giniatullin R. (2020). The Emerging Role of Mechanosensitive Piezo Channels in Migraine Pain. Int. J. Mol. Sci..

[47] Syeda R., Xu J., Dubin A.E., Coste B., Mathur J., Huynh T., Matzen J., Lao J., Tully D.C., Engels I.H. (2015). Chemical Activation of the Mechanotransduction Channel Piezo1. Elife.

[48] Coste B., Mathur J., Schmidt M., Earley T.J., Ranade S., Petrus M.J., Dubin A.E., Patapoutian A. (2010). Piezo1 and Piezo2 Are Essential Components of Distinct Mechanically Activated Cation Channels. Science.

[49] Zhang R., Tomida M., Katayama Y., Kawakami Y. (2004). Response Durations Encode Nociceptive Stimulus Intensity in the Rat Medial Prefrontal Cortex. Neuroscience.

[50] Simard J.M., Sheth K.N., Kimberly W.T., Stern B.J., del Zoppo G.J., Jacobson S., Gerzanich V. (2014). Glibenclamide in Cerebral Ischemia and Stroke. Neurocrit. Care.

[51] Rungta R.L., Choi H.B., Tyson J.R., Malik A., Dissing-Olesen L., Lin P.J.C., Cain S.M., Cullis P.R., Snutch T.P., MacVicar B.A. (2015). The Cellular Mechanisms of Neuronal Swelling Underlying Cytotoxic Edema. Cell.

[52] Leinonen V., Vanninen R., Rauramaa T. (2018). Raised Intracranial Pressure and Brain Edema. Handbook of Clinical Neurology.

[53] (2016). GBD 2015 Mortality and Causes of Death Collaborators Global, Regional, and National Life Expectancy, All-Cause Mortality, and Cause-Specific Mortality for 249 Causes of Death, 1980-2015: A Systematic Analysis for the Global Burden of Disease Study 2015. Lancet.

[54] Qu S., Hu T., Qiu O., Su Y., Gu J., Xia Z. (2020). Effect of Piezo1 Overexpression on Peritumoral Brain Edema in Glioblastomas. AJNR Am. J. Neuroradiol..

[55] Wang Y.-Y., Zhang H., Ma T., Lu Y., Xie H.-Y., Wang W., Ma Y.-H., Li G.-H., Li Y.-W. (2019). Piezo1 Mediates Neuron Oxygen-Glucose Deprivation/Reoxygenation Injury via Ca2+/Calpain Signaling. Biochem. Biophys. Res. Commun..

[56] Zhao W., Wei Z., Xin G., Li Y., Yuan J., Ming Y., Ji C., Sun Q., Li S., Chen X. (2021). Piezo1 Initiates Platelet Hyperreactivity and Accelerates Thrombosis in Hypertension. J. Thromb. Haemost..

[57] Cao Z., Balasubramanian A., Marrelli S.P. (2014). Pharmacologically Induced Hypothermia via TRPV1 Channel Agonism Provides Neuroprotection Following Ischemic Stroke When Initiated 90 Min after Reperfusion. Am. J. Physiol. Regul. Integr. Comp. Physiol..

[58] Muzzi M., Felici R., Cavone L., Gerace E., Minassi A., Appendino G., Moroni F., Chiarugi A. (2012). Ischemic Neuroprotection by TRPV1 Receptor-Induced Hypothermia. J. Cereb. Blood Flow. Metab..

[59] Xie Q., Ma R., Li H., Wang J., Guo X., Chen H. (2021). Advancement in Research on the Role of the Transient Receptor Potential Vanilloid Channel in Cerebral Ischemic Injury (Review). Exp. Ther. Med..

[60] Lee J., Saloman J.L., Weiland G., Auh Q.-S., Chung M.-K., Ro J.Y. (2012). Functional Interactions between NMDA Receptors and TRPV1 in Trigeminal Sensory Neurons Mediate Mechanical Hyperalgesia in the Rat Masseter Muscle. Pain.

[61] Borbiro I., Badheka D., Rohacs T. (2015). Activation of TRPV1 Channels Inhibits Mechanosensitive Piezo Channel Activity by Depleting Membrane Phosphoinositides. Sci. Signal.

[62] Kimmelman J., Mogil J.S., Dirnagl U. (2014). Distinguishing between Exploratory and Confirmatory Preclinical Research Will Improve Translation. PLoS Biol..

[63] Reglodi D., Tamás A., Somogyvári-Vigh A., Szántó Z., Kertes E., Lénárd L., Arimura A., Lengvári I. (2002). Effects of Pretreatment with PACAP on the Infarct Size and Functional Outcome in Rat Permanent Focal Cerebral Ischemia. Peptides.

[64] Kuts R., Frank D., Gruenbaum B.F., Grinshpun J., Melamed I., Knyazer B., Tarabrin O., Zvenigorodsky V., Shelef I., Zlotnik A. (2019). A Novel Method for Assessing Cerebral Edema, Infarcted Zone and Blood-Brain Barrier Breakdown in a Single Post-Stroke Rodent Brain. Front Neurosci..

